# Management of a patient with colon cancer and rotor syndrome: A case report

**DOI:** 10.3892/ol.2013.1766

**Published:** 2013-12-19

**Authors:** DENIZ ARSLAN, FATMA AVCI, ALPARSLAN MERDIN, SEYDA GUNDUZ, HASAN SENOL COSKUN

**Affiliations:** Department of Medical Oncology, Akdeniz University Faculty of Medicine, Antalya 07070, Turkey

**Keywords:** rotor syndrome, colon carcinoma, chemotherapy, oxaliplatin, capecitabine

## Abstract

Rotor Syndrome (RS) is a rare disease that is autosomal recessive and characterized by asymptomatic jaundice, conjugated hyperbilirubinemia and coproporphyria. RS occurs as a result of a complete lack or partial defect of organic anion transporter polypeptides (OATPs) on the basolateral surface of hepatocytes. OATPs facilitate the excretion of bilirubin and organic anions from the liver to the bile. To the best of our knowledge, there is no information in the literature relating to the treatment of a patient with colon cancer and RS. The present study aimed to discuss the systematic chemotherapy that is used and the effects on a 45-year-old patient who had RS with asymptomatic jaundice and was diagnosed with colon adenocarcinoma following surgery. The patient was administered oxaliplatin in combination with capecitabine. The patient’s biluribin level increased after one week. Capecitabine treatment was interrupted and the patient was administered oxaliplatin monotherapy. No significant toxicity was observed during that period. At the latest follow-up the patient did not exhibit any progression.

## Introduction

Rotor Syndrome (RS) is a rare autosomal recessive syndrome that is characterized by conjugated hyperbilirubinemia and coproporphyria (1–4). The main clinical manifestation is non-pruritic jaundice. When compared with Dubin Johnson Syndrome, the pigmentation in the liver is not observed in RS, although the two conditions exhibit similar clinical findings. Histopathological liver biopsies of these patients have a normal appearance. While the etiology of RS is not well known, it has been suggested that the condition occurs as a result of a complete lack or partial defect of organic anion transporter polypeptides (OATPs) on the basolateral surface of hepatocytes. The bile ducts are not viewable in hepatobiliary scintigraphy due to an impaired liver uptake of radioactive material (5,6). No information is available in the literature with regard to the association between colon cancer and RS. The present study aimed to discuss the applied systematic chemotherapy and its effect on a case of RS with developed colon cancer.

## Case report

A 47-year-old male patient with a diagnosis of operative-rectal adenocarcinoma was admitted to the Department of Medical Oncology (Akdeniz University Faculty of Medicine, Antalya, Turkey) in order to undergo adjuvant chemotherapy. Upon physical examination, the whole body and sclerae were icteric. A 5–6-cm long scar track and colostomy was present on the anterior abdominal wall due to prior surgery. Other systemic examinations were normal. The patient was evaluated as T2N1M1 (stage four) according to the TNM staging system. Upon pathological determination of colon serosal invasion, three regional lymph node metastases were determined using positron emission tomography (PET). Hypermetabolic nodules (1.2 cm) on the posterior segment of the lower lobe accounted for two of the node metastases, while the remaining node was situated on the anterior portion of the upper lobe (1 cm). When investigating the blood and biochemical parameters of the patient, a total bilirubin level of 6.7 mg/dl and a direct bilirubin level of 5 mg/dl were observed. The transaminase, γ-glutamyl transferase, alkaline phosphatase, blood urea nitrogen (BUN) and creatine levels were determined to be within the normal ranges. Due to the conjugated hyperbilirubinemia the patient was diagnosed with RS subsequent to being admitted for jaundice. Treatment with ursodeoxycholic acid was administered for a long period of time. At the multidisciplinary oncology council, a metastasectomy was decided upon for the metastatic foci on the left side of the lung. The surgical margins were determined as positive when the metastasectomy materials were examined. The patient provided written informed consent.

**Figure 1:**
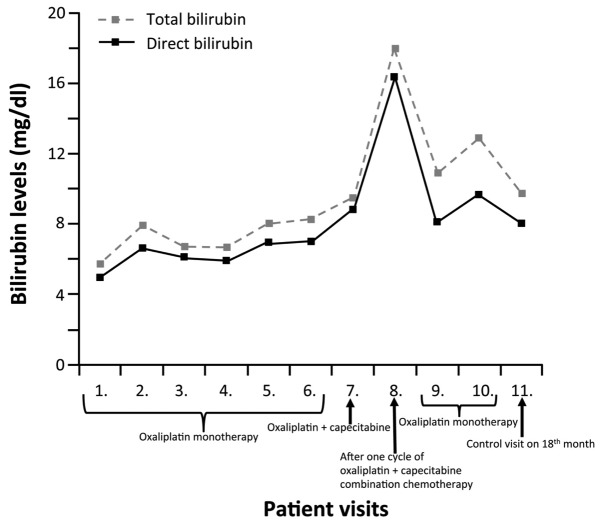
Changes in the total blood bilirubin and direct bilirubin levels of the patient.

Conformal radiotherapy was administered at 3,000 cGy to the metastasectomy area and at 5,040 cGy to the pelvis. Six cycles of systemic chemotherapy were administered to the patient following radiotherapy, every three weeks with 85 mg/m^2^/day oxaliplatin. The disease was evaluated as stable as a result of a control PET scan following the sixth cycle. The treatment of the patient was arranged as a combination chemotherapy and consisted of 1,000 mg/m^2^ capecitabine twice a day for two weeks in combination with 85 mg/m^2^/day oxaliplatin every three weeks. The patient’s biluribin level increased after one week. Capecitabine treatment was interrupted, as the total blood bilirubin level, which was drawn two weeks following the first cycle of chemotherapy using oxaliplatin + capecitabine, was determined as 18 mg/dl. In the liver and bile tracts, ultrasonography was performed due to the total blood bilirubin levels. The intrahepatic bile tracts were evaluated as normal in the patient, who was diagnosed with grade I hepatosteatosis, which was not a focal lesion in the liver parenchyma. Two more cycles of oxaliplatin monotherapy were administered to the patient. The changes in the total blood bilirubin levels of the patient are shown in Fig. 1. PET was administered to the patient, who underwent a total of nine cycles of oxaliplatin therapy. Pathological findings were not determined in the colonoscopy. However, hypermetabolic wall thickening was determined in the right lower quadrant of the gut wall. It was decided at the multidisciplinary oncology council that the post-operative and metastatic situations of the patient, who had tumor markers in the normal range during the pre-operative period, should be followed up without treatment. The patient was followed up without treatment or progression for 18 months.

## Discussion

RS is a rare syndrome that is characterized by familial conjugated hyperbilirubinemia. The etiology of RS is not well known, however, it has been suggested that RS occurs due to the partial defect or lack of OATPs, including OATP1B1 and OATP1B3, which are located on the basolateral surface of hepatocytes and function to excrete organic anions, bile acids and bilirubin bile sinusoids (5,6). There is no information in the literature with regard to patients with RS and colon carcinoma. In one previous study, it was determined that the *de novo* expression of the SLC21 gene, which encodes OATP proteins, was elevated in breast, colon, pancreas, stomach and prostate cancers (7). It is indicated in the literature that various single gene polymorphisms on the OATP1 gene cause an elevation in the drug levels in the tissues and blood by reducing the elimination of certain drugs, including statins, methotrexate and irinotecan (8).

There is no information with regard to the use of 5-flourourasil (5-FU) and capecitabine for the treatment of patients with RS and colon carcinoma. 5-FU and capecitabine are antimetabolite chemotherapeutic drugs that are analogs of pyrimidine and are metabolized and excreted in the liver. However, oxaliplatin is primarily eliminated from the kidneys (9). The present study determined the presence of a significant increase in the total blood bilirubin levels in the patient following the administration of oxaliplatin chemotherapy. The patient tolerated the oxaliplatin chemotherapy well. However, following the capecitabine chemotherapy, a three-fold increase in the basal total blood bilirubin levels (basal total blood bilirubin of the patient was 6 mg/dl) was observed and the Eastern Cooperative Oncology Group performance increased from 0 to 3. Therefore, capecitabine chemotherapy is not the right agent to administer to patients with RS and colon carcinoma.

In conclusion, there is no information in the literature with regard to RS with colon carcinoma. Oxaliplatin was an applicable agent for use in the patient with RS and colon carcinoma as there were no side-effects in determining the total blood bilirubin levels and was well tolerated. The patient has been followed up for 18 months without treatment or progression.
